# XCOVNet: Chest X-ray Image Classification for COVID-19 Early Detection Using Convolutional Neural Networks

**DOI:** 10.1007/s00354-021-00121-7

**Published:** 2021-02-24

**Authors:** Vishu Madaan, Aditya Roy, Charu Gupta, Prateek Agrawal, Anand Sharma, Cristian Bologa, Radu Prodan

**Affiliations:** 1 Lovely Professional University, Phagwara, Punjab India; 2 Bhagwan Parshuram Institute of Technology, New Delhi, India; 3 University of Klagenfurt, Klagenfurt, Austria; 4 Mody University of Science and Technology, Laxmangarh, Rajasthan India; 5 Babes-Bolyai University, Cluj-Napoca, Romania

**Keywords:** Coronavirus, SARS-COV-2, COVID-19 disease diagnosis, Machine learning, Image classification

## Abstract

COVID-19 (also known as SARS-COV-2) pandemic has spread in the entire world. It is a contagious disease that easily spreads from one person in direct contact to another, classified by experts in five categories: asymptomatic, mild, moderate, severe, and critical. Already more than 66 million people got infected worldwide with more than 22 million active patients as of 5 December 2020 and the rate is accelerating. More than 1.5 million patients (approximately 2.5% of total reported cases) across the world lost their life. In many places, the COVID-19 detection takes place through reverse transcription polymerase chain reaction (RT-PCR) tests which may take longer than 48 h. This is one major reason of its severity and rapid spread. We propose in this paper a two-phase X-ray image classification called XCOVNet for early COVID-19 detection using convolutional neural Networks model. XCOVNet detects COVID-19 infections in chest X-ray patient images in two phases. The first phase pre-processes a dataset of 392 chest X-ray images of which half are COVID-19 positive and half are negative. The second phase trains and tunes the neural network model to achieve a 98.44% accuracy in patient classification.

## Introduction

COVID-19 is an infectious and contagious disease pandemic that spread over 200 countries in the past year^1^. Meanwhile, COVID-19 has become a serious health threat for the entire world that causes respiratory problems, heart infections, and even death. This virus first reported in a human being in December 2019 in Wuhan, China^2^ rapidly crossed the continent borders due to intensive travelling among countries. COVID-19 has had an adverse impact on the world economy too. Research studies found out that the COVID-19 virus badly affects the lungs and promptly mutates before the patient receives any diagnosis led medication [1, 2]. The situation becomes more severe when the symptoms match the normal flu, as in the South East and Central Asia cases. Experts found out that the incubation period of COVID-19 virus is approximately 1 week [3]. This observation is crucial because the infected patient acts as a virus carrier during this period and unintentionally transmits it. Due to its rapid contagious nature, its spread is much faster than its detection. Machine learning methods [4–6] are very popular in healthcare applications. There are various methods used to detect the presence of a COVID-19 virus in patients, such as RTPCR test [7], X-ray imaging [8, 9], computed tomography (CT) scan [10], rapid antigen [11], serological test [12].

### Motivation

While RTPCR [7] is by far the most effective way of COVID-19 detection. this method is very time consuming (taking hours to even days) and requires special kits that may not be available in remote regions of a country due to geological, social and economic barriers. On the contrary, the rapid antigen test looks for the presence of antigens of the virus from a nasal swab but suffers from higher rate of false negatives. The serological test looks for the antibodies produced by the immune system against the virus from the blood sample of the patient. However, it only checks the IgM and IgG antibodies during or after recovery and does not help in early virus detection. CT scan and X-ray scans, both use invisible ranges of electro-magnetic spectrum to detect any kind of anomaly, used for early detects and have high clinical relevance. In this paper, we found out that the chest X-ray tests are economically affordable and the results are relatively easy to use. Chest X-ray tests are easily available, have portable versions, and a low risk of radiation. On the other hand, CT scans have high risk of radiation, are expensive, need clinical expertise to handle and are non-portable. This makes the use of X-ray scans more convenient than CT scans.

### Approach

Considering the advantages of X-ray tests, we propose a novel model called XCOVNet that uses a convolution of positive $\mathrm {COVID}^+$ and negative $\mathrm {COVID}^-$ chest X-ray images to train a network and detect COVID-19 viruses in early infection stages. The developed a convolutional neural network (CNN) categorize the chest X-ray images of patients as positive $\mathrm {COVID}^+$ or negative $\mathrm {COVID}^-$. Our XCOVNet model uses a CNN with the *Adam* optimizer and a learning rate 0.001. It does not require any feature selection method and uses a handcrafted seed dataset for CNN local and global features with 196 of $\mathrm {COVID}^+$ patient chest X-ray images and 196 of $\mathrm {COVID}^-$ images. The recorded X-ray images (positive and negative) belong to different domains and have multiple views of the same scan to minimize the bias towards a particular class. As the segmentation of pneumonia related images was very difficult in earlier approaches, the proposed XCOVNet model based on computerised automated detection can understand the features more efficiently and detect COVID-19 faster than other classical learning methods. Our trained CNN comprises three convolutional layers with the kernel size of $3 \times 3$ followed by a rectified linear unit (ReLU) activation function which takes input images of size $224 \times 224 \times 3$. We trained, tested and validated the proposed XCOVNet system and achieved an accuracy of 98.44% in classifying chest X-ray images.

### Contribution

Model training to predetermined images (having seed data as well), affects the bias, variance trade-off and results in under-fitting and over-fitting of the model. To reduce these drawbacks, we tuned the XCOVNet model on four sets with different training and testing data ratios, with the F1 score ranging from 89.26 to 97.94%, unlike other state-of-the-art works (see Table 5 in Sect. 4). We further tuned XCOVNet model in three different ways, unlike earlier approaches:

1. We trained the entire architecture;
2. We trained some layers and froze the others;
3. We froze the entire architecture.

While existing studies focused on deep learning, they do not pay much attention to model tuning. The advantage of the proposed XCOVNet model is the proper use of structural deep network, improved parameter value selection and refined succinct boundary conditions. In this paper, we empirically experimented with a different dataset with twenty distinct parameters (see Table 3 in Sect. 4), where the learning process did the tuning exercise for effective selection and adjustment of the parameters. Table 6 validates the proposed XCOVNet model that outperforms existing state-of-the-art methods in terms of accuracy. Finally, the tuning and training give a plausible configuration of our XCOVNet model, which gives insights to the critical behaviour of the parameters used in its architecture.

### Outline

The paper has five sections. Section 2 highlights the related work. Section 3 describes the proposed XCOVNet method comprising the two phases with emphasis on the CNN model development, together with the dataset used in its evaluation. Section 4 evaluates the proposed model results and compares the results with the state-of-the-art research. Section 5 concludes the paper and highlights future work.

## Related work

This section reviews various important COVID-19 detection methods.

### CNN and medical image classification

CNN is one of the most impressive deep neural networks that incorporates numerous hidden layers performing convolution and sampling to extricate low to significant levels of input data. This network indicated a high effectiveness in various zones, especially in computer vision [13]. CNN can incorporate various convolutional layers, where the sources of data and output of next convolutional layers are feature vectors. There are different channels convoluted with the information in every layer. The profundity of the generated feature maps is comparable to the quantity of filters applied in the convolution activity [14]. Pasa et al. [15] proposed simple CNN architectures for chest X-ray to optimize the tuberculosis visualization.

### Pneumonia and COVID-19

Pneumonia can be brought by various kinds of bacteria and viruses. It is tedious and tiring for general radiologists in the network emergency clinics to peruse a high volume of chest X-beam pictures to recognize an unobtrusive COVID-19 contaminated pneumonia from other networks. Radiologists increasingly pursue this clinical test during this pandemic, as there are numerous likenesses between pneumonia tainted by COVID-19 and different sorts of bacteria or viruses [16]. Computer scientists already experimented with various intelligent methods to detect the COVID-19 infection well in advance. Ultraviolet scans effectively detect the patches that occur in lungs called as ground glass opacity [17, 18]. This is in a continuation stage where the patients suffer from progressive pneumonia and can lead to acute respiratory distress syndrome. As the infection of this virus mainly attacks the patients’ respiratory system [19], researchers mainly focused on detecting the disease level by finding the infection level using chest X-ray images [20], chest CT scan images [21] or cough sound recognition [22]. Researchers implemented various deep learning and computer vision methods to classify the patients of this infectious disease from healthy patients [15, 23, 24]. Accordingly, numerous deep learning models in the late literature aim to distinguish and order COVID-19 cases. Although some deep learning-based CNN models are applied to CT images, more studies applied CNN models detect and classify COVID-19 cases using chest X-ray images, including DarkCovidNet [25] and COVIDX-Net [26]. Chest X-ray radiography is commonly used in clinical practice due to its low cost, low radiation dose, easy-to-operate and wide accessibility advantages in the general or community hospitals [27]. Medical images found reliable and played important role in effectively controlling COVID-19 spread and treating patients to reduce mortality rate [28].

### COVID-19 diagnosis using chest CT scans

Ai et al. [29] found out that chest CT scans help in detecting the COVID-19 infections faster than RTPCR tests. Moreover, repetitive scanning may be useful to monitor the recovery or infection severity of patients. Li et al. [30] developed a deep learning-based COVNet model to categorise the CT scan images among COVID-19, pneumonia and other lung diseases. Wang et al. [21] implemented another deep learning method on chest CT scan images for graphical pathogenic tests to detect COVID-19. Li and Xia [31] used chest CT images of COVID-19 patients and compared them with non-infected patients to identify the exact discrimination in the infection patterns and reduce the false positive rate in their results.

### COVID-19 diagnosis using chest X-ray images

Sethy et al. [32] developed a deep leaning-based model to detect a COVID-19 infection using X-ray images and achieved an up to 95.38% accuracy. Similarly, Apostolopoulos and Mpesiana [33] proposed another deep leaning model on X-ray images to predict COVID-19 with a maximum of 98% accuracy. Asnaoui et al. [34] applied an inception-ResNetV2 model on chest CT scans and X-ray images and achieved a highest 92.18% accuracy classification in bacterial COVID-19, normal and pneumonia cases. Ozturk et al. [25] implemented two deep learning-based models for binary classification (COVID-19 and non-COVID-19) and ternary classification (COVID-19, non-COVID-19 and pneumonia) and achieved maximum accuracy of 98.08% and 87.02%, respectively. Azemin et al. [20] proposed a deep learning-based COVID-19 prediction model to classify the chest X-ray images into COVID-19 and non-COVID-19 ones with a maximum accuracy of 71.9%. Abiyev and Ma’aitah [35] classified the chest X-rays images using CNN for the diagnosis of chest diseases. Heidari et al. [23] demonstrated radio-graphic chest images to detect COVID-19 and assess disease severity. They collected around 8474 chest X-ray dataset images and divided in three groups: non-pneumonia, COVID-19 infected pneumonia and other community-acquired non-COVID-19 infected pneumonia cases. Oh et al. [24] used a statistical method to analyze the potential chest X-ray COVID-19 markers. They worked on a local patch-based approach using lung morphology, mean lung intensity, and the standard deviation of lung intensity in five classes: normal lungs, bacterial pneumonia, viral pneumonia, tuberculosis, and COVID-19 chest X-ray images. Their model achieved a highest classification accuracy of up to 88.9%.

### Summary

Table 1 summarizes the related work and reveals that the deep learning methods are becoming popular to classify the COVID-19 infected cases using the chest CT scan images and chest X-ray images. However, researchers could merely achieve maximum accuracy up to 87% using chest CT scan image classification. In contrast, the chest X-ray images appear to more capable to predict the infection with better accuracy. As the authors of the state-of-the-art works did not clearly explain their experimental design, it is difficult for us to test their models on our dataset. Based on these observations, we proposed a CNN-based model called XCOVNet to detect COVID-19 infections using chest X-ray images of suspected patients.

**Table 1:** Related work summary

Related work	Technique	Dataset	Accuracy (%)
Wang et al. [21]	Inception transfer learning	Chest CT scan	85.2
Sethy et al. [32]	Deep learning	Chest X-ray images	95.38
Azemin et al. [20]	Deep learning	Chest X-ray images	71.9
Oh et al. [24]	Statistical method	Chest X-ray images	88.9
XCOVNet model	CNN	Chest X-ray images	98.44

## XCOVNet method

**Fig. 1:**
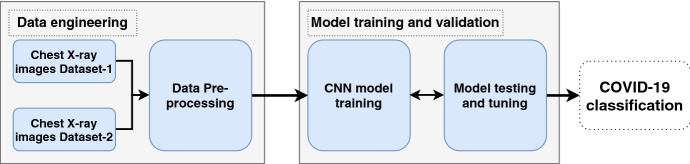
XCOVNet model phases

This section describes XCOVNet method consisting of two phases, illustrated in Fig. 1: (i) data engineering and (ii) model training and validation.

### Data engineering

We use two chest X-ray image datasets in our method, summarized in Table 2.

Dataset-1 [36] contains total of 950 X-ray images^3^ labeled with more than fifteen types of disease findings such as: pneumocystis, streptococcus, klebsiella, legionella, SARS, lipoid, varicella, mycoplasma, influenza, herpes, aspergillosis, nocardia, COVID-19, tuberculosis and others. This image dataset contains anteroposterior (front to back), front postero-anterior (back to front) and lateral (side) X-ray image views. Front postero-anterior images give clear lung representations, therefore we selected 196 $\mathrm {COVID}^+$ pre-processed chest X-ray images labelled with front view for our experiments and removed the rest.

**Fig. 2:**
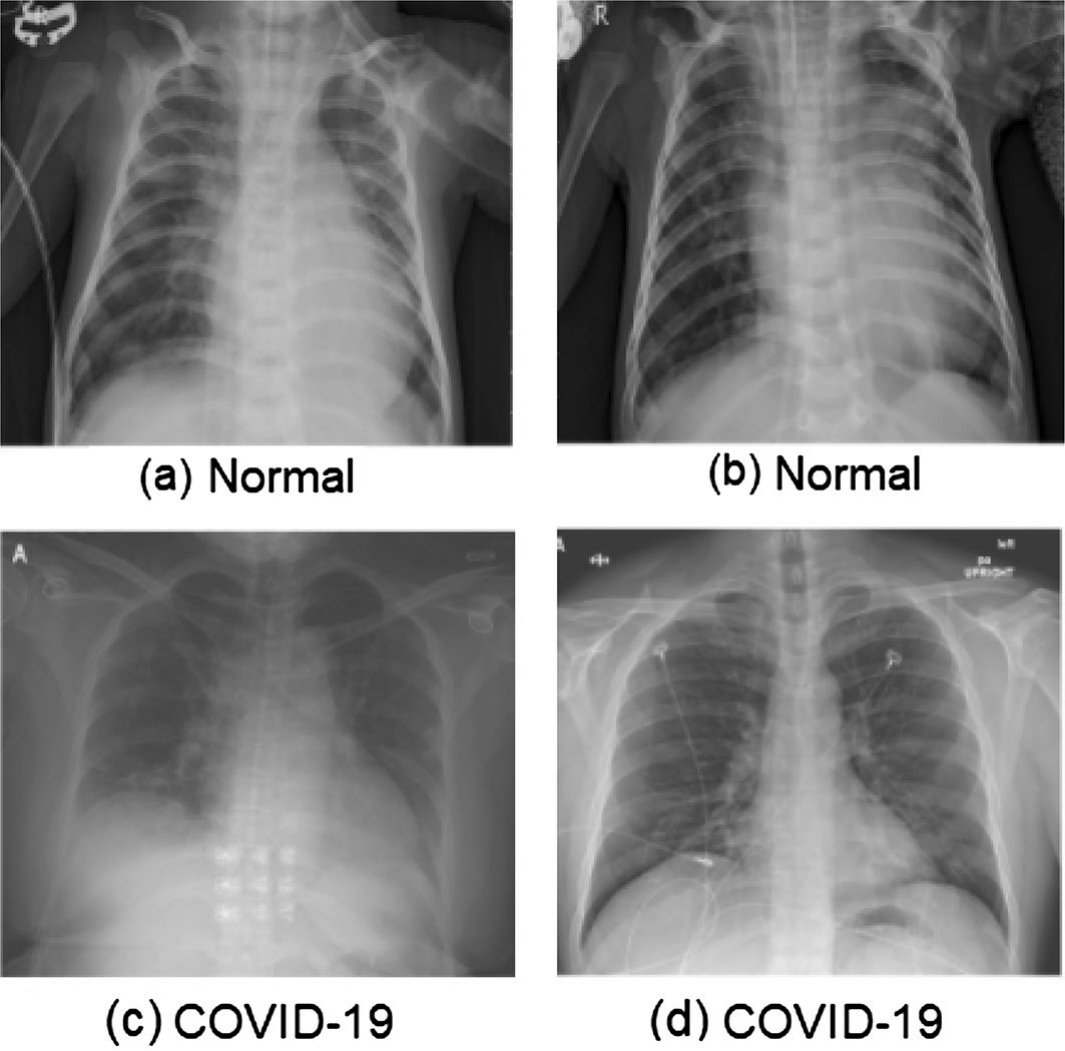
X-ray image samples of COVID-19-infected and healthy (*i.e.*, normal) patients

Dataset-2 contains total 5856 chest X-ray images labeled in three categories: normal, viral pneumonia, and bacterial pneumonia. All X-ray images have a front posteroanterior view. We randomly selected 196 X-ray images of normal category and labelled them as $\mathrm {COVID}^-$ image type. The reason for this selection was to keep the data unbiased and balanced by keeping $\mathrm {COVID}^+$ and $\mathrm {COVID}^-$ data size equal. We performed four image pre-processing steps to reduce the noise: (i) re-scaling, (ii) shearing, (iii) zooming, and (iv) horizontal flip. Finally, we reduced the pre-processed image size to $224 \times 224 \times 3$ and made them uniform before applying model training (Fig. 2).

**Table 2:** Dataset description

X-ray image type	X-ray front posteroanterior view	Dataset-1 image count [36]	Dataset-2 image count [37]
COVID-19 positive	$\checkmark$	196	–
	$\times$	388	–
COVID-19 negative	$\checkmark$	–	1583
	$\times$	–	–
Other disease	–	366	4273

### XCOVNet CNN architecture

**Fig. 3:**
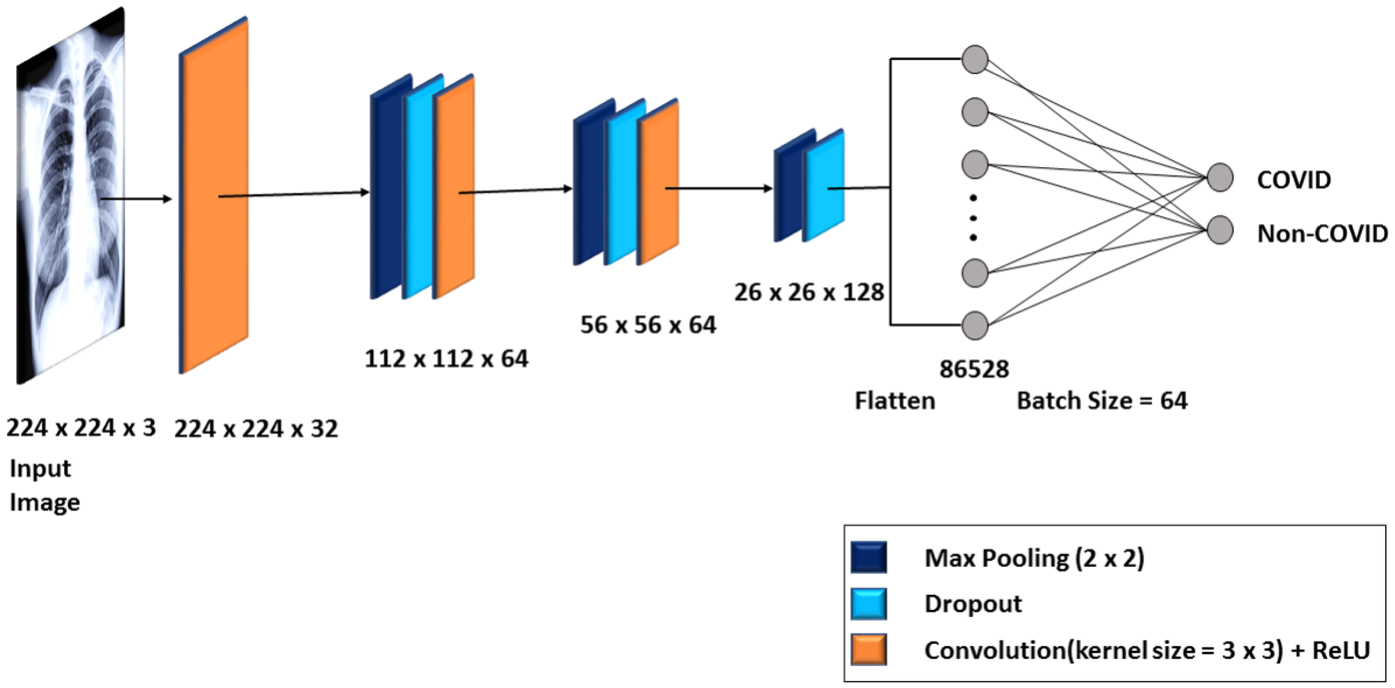
XCOVNet CNN architecture

**Fig. 4:**
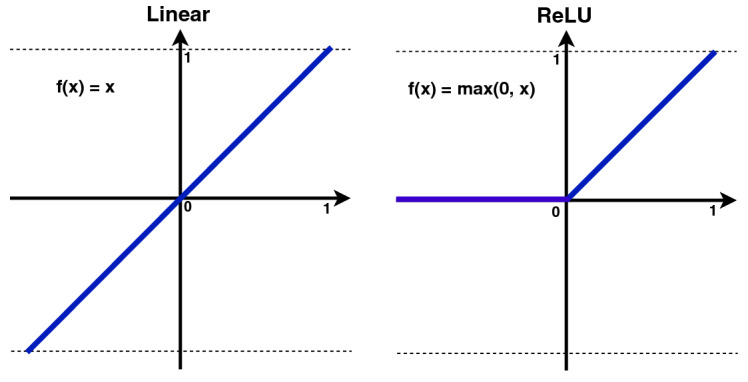
Linear and ReLU activation function

We used a layered sequential model architecture for X-ray image classification with four convolutional layers displayed in Fig. 3. The first convolutional layer has 32 filters, the second layer has 64 filters, the third layer has 64 filters and the last layer has 128 filters. The number of filters corresponds to number of features the network can extract at each layer. We gradually increased the number of filters in the proposed network because the lower layers detect features in a very small part of the image and learn a hidden pattern during the network training. The receptive field of the CNN layer architecture increases with its depth in the network. This means that by increasing the number of layers, the network extracts the features from the larger part of the original picture, as the deeper layers in the network will detect higher level features. We fixed the default kernel size to $3 \times 3$ at the convolutional layer and applied a non-linear ReLU activation function. Figure 4 clearly shows that the ReLU curve is half rectified, unlike the linear activation function. This means that ReLU returns zero as output value for all negative input values and represented as:

$$\begin{aligned} f(y)={\left\{ \begin{array}{ll} 0, &{} \text {if y<0}.\\ y, &{} \text {otherwise}. \end{array}\right. } \end{aligned}$$

If the value of *y* is less than 0 then value of *f*(*y*) will be zero otherwise output will be *y*.

Similarly, we used three max-pooling layers and kernel window of size $2 \times 2$ with the increased number of filters in each layer to contain the more complex image patterns in training network. Table 3 summarizes the proposed CNN model parameters, used to classify the chest X-ray dataset.

**Table 3:** CNN model parameters for chest X-ray image dataset

Parameters	Value
Number of convolutional layers	4
Maximum number of pooling layers	3
Number of filters at convolutional layer	32, 64, 128
Kernel window size at convolutional layer	$3 \times 3$
Kernel window size at maximum pool layer	$2 \times 2$
Number of strides at convolutional layer	1
Number of strides at maximum pool layer	1
Number of neurons in output layer	2
Learning rate	0.001
Number of epochs	50
Number of iterations (per epoch)	8
Input image size	$224 \times 224 \times 3$
Number of input attributes	150, 528
Number of output attributes	2
Training data size	294
Testing data size	98
Training and testing data size ratio	75% / 25%
Activation function (before and after maximum pooling layer)	ReLU
Batch size	32
Optimizer	Adam

We implemented proposed CNN model on the selected datasets with 196 positive and 196 negative COVID-19 images. We trained the model and tuned it using different learning parameters and training and testing dataset distributions. We experimented with three CNN architectures:

1. *CNN model-1* with a maximum pool size of $2 \times 2$ and one stride;
2. *CNN model-2* with a maximum pool size of $2 \times 2$ and two strides;
3. *CNN model-3* with a maximum pool size of $3 \times 3$ and three strides.

We performed four training-testing data splits on each CNN architecture: (i) 60–40%, (ii) 70–30%, (iii) 75–25%, and (iv) 80–20%. We found out that the 75–25% training and testing data split with CNN model-1 achieves the highest performance compared to other options. We explain the detailed performance analysis in Table 5 in Sect. 4.2. Finally, we trained our proposed CNN model with 294 chest X-ray images (147 being $\mathrm {COVID}^+$ and 149 being $\mathrm {COVID}^-$) and tested it using a set of 98 images (49 of $\mathrm {COVID}^+$ and 49 $\mathrm {COVID}^-$ images).

## Results and analysis

### Evaluation metrics

We used four parameters, *true positive (TP)*, *true negative (TN)*, *false positive (FP)* and *false negative (FN)* displayed in Table 4, to evaluate the overall performance of proposed CNN model on four performance metrics: *accuracy*, *precision*, *recall* and *F1-score* [38].

**Table 4:** Evaluation parameters

Parameter	Predicted value	Actual value
True positive	Yes	Yes
True negative	No	No
False positive	Yes	No
False negative	No	Yes

Accuracy is a measure the model correctness, defined as the ratio between the number of correct predictions and the total number of predictions:

$$\begin{aligned} \mathrm {Accuracy} = \frac{\mathrm{TP} + \mathrm{TN}}{\mathrm{TP} + \mathrm{TN} + \mathrm{FP} + \mathrm{FN}}. \end{aligned}$$

Precision measures the positive predicted value, as the ratio between the number of correct positive predictions to the total number of positive predictions:

$$\begin{aligned} \mathrm {Precision} = \frac{\mathrm{TP}}{\mathrm{TP} + \mathrm{FP}}. \end{aligned}$$

Recall measures the sensitivity of the model, defined as the ratio between the number of correct positive predictions to the total number of correctly predicted results:

$$\begin{aligned} \mathrm {Recall} = \frac{\mathrm{TP}}{\mathrm{TP} + \mathrm{FN}}. \end{aligned}$$

F1-score measures the testing accuracy of the model, defined as the harmonic mean of the precision and the recall:

$$\begin{aligned} {F1 {-}\mathrm{score}} = 2 \cdot \frac{\mathrm{Precision} \cdot \mathrm{Recall}}{\mathrm{Precision} + \mathrm{Recall}}. \end{aligned}$$

### Results

We took 60–40%, 70–30%, 75–25% and 80–20% of training and testing data splits on all three models and examined their performances in terms of accuracy, precision, recall and F1-score, introduced in Sect. 4.1. Table 5 shows the detailed performance analysis of the proposed XCOVNet model on the three CNN architectures and presents the evaluation metrics for the four training and testing dataset splits. Thus, we analysed overall performance of total twelve models ($3 \times 4$) and found out that overall, CNN model-1 performs best on four performance metrics followed by CNN model-3 and CNN model-2. The CNN model-2 gives the least consistent results for different training-testing dataset splits. We therefore selected CNN model-1 foe the proposed XCOVNet model.

Our XCOVNet model produces the best results with a maximum accuracy of $98.44\%$ using the 75–25% training-testing split and the lowest accuracy of $97.43\%$ using the 80–20% split. The model achieves the highest precision of $99.29\%$ with the 70–30% split, followed by 75–25%, 80–20% and 60–40%. The proposed XCOVNet model achieves the highest F1-score of $97.94\%$ using 75–25% split and lowest F1-score $89.26\%$ using 70–30% split. XCOVNet produces maximum recall value of $99.48\%$ using the 60–40% split followed by 80–20%, 75–25% and 70—30%.

We conclude from these results that the proposed XCOVNet model achieves the better performance on the chest X-ray image dataset (on all evaluation metrics) using the 75–25% training-testing split on CNN model-1.

**Table 5:** XCOVNet model evaluation results

Training-testing data ratio (%)	Accuracy (%)	Precision (%)	Recall (%)	F1-Score (%)
CNN model-1 (Maximum pool size: $2 \times 2$; stride: 1)				
$60-40$	98.36	83.69	99.48	90.90
$70-30$	97.45	99.29	80.61	89.26
$75-25$	98.44	98.45	97.44	97.94
$80-20$	97.43	97.46	97.95	97.70
CNN model-2 (Maximum pool size: $2 \times 2$; stride: 2)				
$60-40$	95.56	98.30	46.93	63.88
$70-30$	94.91	95.52	97.95	96.72
$75-25$	98.37	98.48	90.30	94.90
$80-20$	98.71	97.95	69.89	82.28
CNN model-3 (Maximum pool size: $3 \times 3$; stride: 3)				
$60-40$	97.46	96.50	98.46	97.47
$70-30$	98.30	98.39	54.08	70.19
$75-25$	97.95	92.78	98.46	95.54
$80-20$	96.15	87.24	69.89	93.18

### Related work comparison

We compare our work with four related state-of-the-art methods, summarized in Table 6.

Butt  et al. [39] experimented 528 chest CT scan images out of which, 189 images are of $\mathrm {COVID}^+$ patients while the rest 339 images are of other disease patients. They used CT scan images of size $60 \times 60 \times 3$ and applied two three-dimensional CNN classification models: *(i)* Resnet-18 and *(ii)* Resenet+location-attention model with 1200 epochs and achieved 86.7% accuracy only. They did not share their training model parameters.

Wang  et al. [21] used chest CT scan images and resized them $636 \times 533$ pixels. They implemented inception transfer learning on 1065 chest CT scan images with 325 $\mathrm {COVID}^+$ patients images on 15000 epochs with a very high training loss of 0.6 and claimed 85.2% accuracy.

Sethy  et al. [32] used 381 patients chest X-ray images with three equal size of image data for COVID-19, pneumonia and healthy persons. They applied a ResNet50+SVM classification in their experiments and claimed to achieve maximum 95.33% accuracy. They did not explain the parameters and network specifications in detail.

Apostolopoulos and Mpesiana  [33] re-scaled the X-Ray images to a size of $200 \times 266$. They applied deep CNN with ResNet and inception method on chest X-ray images on default common hyper-parameters and their values. They used ReLU activation function and Adam optimizer and trained the network with two hidden layers, one dropout and ten epochs and achieved maximum 97.82% accuracy.

XCOVNet uses CNN classification on 392 chest X-ray images (equally distributed between $\mathrm {COVID}^+$ and $\mathrm {COVID}^-$) and achieved a 98.44% accuracy. Unfortunately, the authors of the state-of-the-art works did not sufficiently detail their experimental parameters and, therefore, it becomes hard to test their models on our dataset. Although our proposed XCOVNet model achieved encouraging results, it also has some limitations. Firstly, we used a publicly available dataset of 392 cases, including 192 $\mathrm {COVID}^+$. Due to the diversity in COVID-19 case symptoms, the robustness of our proposed work needs further testing and validation using other diverse and larger image datasets. Secondly, the XCOVNet model only investigates and tests with front viewed fixed dimension X-ray images to generate filtered images, which may not be optimal and requires further investigations and extensions.

**Table 6:** XCOVNet comparison with related methods

Related work	Method	Image type	Images	Accuracy
Butt et al. [39]	3-D CNN classification	Chest CT scan	Total: 528 $\mathrm {COVID}^+$: 189	86.7%
Wang et al. [21]	Inception transfer learning	Chest CT scan	Total: 1065 $\text {COVID}^+$: 325	85.2%
Sethy et al. [32]	Resnet50 and SVM classification	Chest X-ray	Total: 381 $\mathrm {COVID}^+$: 127	95.38%
Apostolopoulos and Mpesiana [33]	Deep CNN with ResNet and inception	Chest X-ray	Total: 1427 $\mathrm {COVID}^+$: 224	97.82%
*XCOVNet*	CNN classification	Chest X-ray	Total: 392 $\mathrm {COVID}^+$: 196	98.44%

## Conclusion and future work

We developed in this work a model to detect the COVID-19 infection using chest X-ray images. For this purpose, we used a publicly available dataset of 392 positive $\mathrm {COVID}^+$ and negative $\mathrm {COVID}^-$ X-ray patient images. We fixed each input image size to $224 \times 224 \times 3$ and performed CNN training for a accurate classification. We implemented three convolutional layer based models with a kernel size of $3 \times 3$ and achieved a COVID-19 detection accuracy of 98.44%. We also compared our method with several state-of-the-art works [32, 33] limited to a maximum of 98% accuracy.

Although the proposed XCOVNet model classifies the $\mathrm {COVID}^+$ and $\mathrm {COVID}^-$ patients with more than 98% accuracy using chest X-ray images, we still face a serious need to find out the severity level of the infection too. In the future, we intend to perform experiments on chest CT scan image data for COVID-19 detection and combine both the models to identify the severity level. Voice recognition based early COVID-19 infection detection using intelligent methods is also part of our future plans.

## Footnotes

1 https://www.worldometers.info/coronavirus/

2 https://covid19.who.int/

3 https://github.com/ieee8023/covid-chestxray-dataset/tree/master/images
